# Editorial: Protein Phase Separation and Aggregation in (Patho)Physiology of Neurons

**DOI:** 10.3389/fphys.2022.959570

**Published:** 2022-07-04

**Authors:** Dragomir Milovanovic, Silvio O. Rizzoli

**Affiliations:** ^1^ Laboratory of Molecular Neuroscience, German Center for Neurodegenerative Diseases, Berlin, Germany; ^2^ Institute of Neuro- and Sensory Physiology, University Medical Center Göttingen, Göttingen, Germany

**Keywords:** phase separation, neuron, neurodegenerative diseases, synapse, AMPA and NMDA-type receptors, alpha-synuclein, synaptic vesicles, TDP43

Liquid-liquid phase separation (LLPS) is emerging as a major mechanism for the organization of macromolecules in compartments non-limited by a membrane or scaffold (Banani et al., 2017). During the last few years, a surge of studies demonstrated that the spatial and temporal organization of many structures in neurons is based on LLPS. This ever growing array of structures includes the cluster of synaptic vesicles (SVs) (Milovanovic and De Camilli, 2017; Milovanovic et al., 2018), RNA-containing granules (Lin et al., 2015; Molliex et al., 2015), active zones (Wu et al., 2019; McDonald et al., 2020), postsynaptic densities (both excitatory and inhibitory) (Zeng et al., 2016; Bai et al., 2021). At the same time, several observations led to the conclusion that the dysregulation of LLPS in neurons causes protein and organelle aggregation (Shin and Brangwynne, 2017), which is a hallmark of many neurodegenerative diseases.

A collection of papers within this Research Topic discusses the effects of LLPS on the organization of synapses, RNA-binding proteins and the assembly of RNA granules, and the consequences that the aberrant phase separation has on protein aggregation.

The synaptic bouton is one of the best characterized neuronal compartments (Wilhelm et al., 2014; Reshetniak et al., 2020), with numerous proteins (and protein families) combining here to achieve rapid neurotransmitter release and well-regulated SV recycling. SVs form liquid condensates, able to recruit synaptic proteins such as intersectin and alpha-synuclein (Milovanovic et al., 2018; Pechstein et al., 2020; Hoffmann et al., 2021). (Fouke et al.) capitalize on the large reticulospinal synapse of the lamprey to demonstrate that the acute depletion of alpha-synuclein disrupts the SV cluster in a piecemeal fashion into smaller SV clumps. This corroborates with the studies that indicate that the abundance of alpha-synuclein alters the mesoscale organization of SV condensates. Furthermore, at pathologically high concentrations, alpha-synuclein undergoes LLPS on its path to aggregation (Ray et al., 2020). (Brodin et al.) argue that a dual relation exists between alpha-synuclein and the SV phase: alpha-synuclein presumably helps assemble large SV condensates, and, in return, the biochemical milieu within these condensates ensures that alpha-synuclein remains soluble, and is prevented from pathological aggregate formation.

AMPA receptors are known to form nanoclusters at the postsynapse (Choquet and Hosy, 2020), which are further stabilized during prolonged synaptic activity (Opazo et al., 2010). Interestingly, stargazin, an auxiliary subunit of the AMPA receptor, undergoes LLPS with the proteins of the postsynaptic density, which affects the dynamics of both AMPA and NMDA receptors in excitatory synapses (Zeng et al., 2018; Hosokawa et al., 2021). In a review, (Hosokawa and Liu) discuss the relationship between synaptic plasticity, LLPS, and the AMPA receptors at the postsynaptic plasma membrane.

Neurons are highly polarized cells, with the axons of motor neurons being particularly clear examples of the cellular polarization. They can reach lengths of over 1 m, leading to substantial pressure on the transport of organelles and molecular complexes, such as the RNA-containing transport granules. TDP-43 is a nucleic acid-binding protein that is often associated with RNA transport granules in the neuronal cytoplasm and is able to undergo LLPS (Conicella et al., 2016). (Vishal et al.) investigate how the mutations that disrupt LLPS of TDP-43 affect the trafficking of TDP 43-containing granules in axons. The authors show that the conserved alpha-helical domain at the C-terminal region, surrounding FG motifs, tryptophan residues, and RGG motifs all affect the transport of TDP-43 granules in axons, indicating their relevance for the recruitment of motors and adaptor proteins.

The aberrant phase separation of RNA-binding proteins (RNPs) leads to the formation of aggregates, which are a hallmark of Amyotrophic Lateral Sclerosis (ALS) and Frontotemporal Dementia (Blokhuis et al., 2013). (Carey and Guo) review two specific RNPs, TDP-43, and FUS, two molecules that have been implicated in disease, and are known to undergo phase separation. In fact, (Milicevic et al.) argue that LLPS-disrupting mutations are a common feature of many RNPs implicated in the pathology of ALS, such annexin 11, ataxin 2, hnRNPA1, hnRNPA2, and TIA-1. The cause-effect relationship between RNA granule assembly, their association with membrane-bound compartments such as ER, lysosomes or mitochondria, and their response to (oxidative) stress, are all important for the appropriate neuronal physiology (Liao et al., 2019; Amen and Kaganovich, 2021; Trnka et al., 2021). The aberrant phase separation of RNPs induces the loss of their cellular function and also triggers the formation of inclusions that may trap folded proteins and membranes.

Misfolded host prion protein (PrP) forms amyloid fibrils, a hallmark of diseases referred to as Transmissible Spongiform Encephalopathies (TSEs) (Heumüller et al., 2022). (Aguilar et al.) demonstrate that differentiating neuronal cells exposed to an infectious TSE agent can induce a dramatic increase in interferon-beta mRNA as well as a reduction of PrP mRNA and protein levels, implying the interplay between the innate immune response and PrP dynamics in TSE. This exciting finding should be followed by studies that will assess the extent to which LLPS is involved in intracellular agent sequestration, and the potential surveillance mechanisms that can affect the ensuing neurodegeneration.

Overall, the papers within this Research Topic showcase the numerous roles LLPS has in neuronal function, in health and disease, from synaptic organization and dynamics, to RNA trafficking in neurites, or to neuronal pathophysiology. The years ahead promise to shed light on the interactions between biomolecular condensates and the better-studied membrane trafficking processes, as well as how to capitalize on these interactions while tackling neurological and neurodegenerative diseases.
